# Assigning credit where it is due: an information content score to capture the clinical value of multiplexed assays of variant effect

**DOI:** 10.1186/s12859-024-05920-5

**Published:** 2024-09-06

**Authors:** John Michael O. Ranola, Carolyn Horton, Tina Pesaran, Shawn Fayer, Lea M. Starita, Brian H. Shirts

**Affiliations:** 1 Ambry Genetics, Aliso Viejo, CA USA; 2 Department of Genome Sciences, University of Washington, Seattle, WA USA; 3 Brotman Baty Institute, Seattle, WA USA; 4 Department of Laboratory Medicine and Pathology, University of Washington, Seattle, WA USA; 5 Institute for Public Health Genetics, University of Washington, Seattle, WA USA

**Keywords:** Information theory, Variant classification, Genetic information, Summary data, Entropy

## Abstract

**Background:**

A variant can be pathogenic or benign with relation to a human disease. Current classification categories from benign to pathogenic reflect a probabilistic summary of the current understanding. A primary metric of clinical utility for multiplexed assays of variant effect (MAVE) is the number of variants that can be reclassified from uncertain significance (VUS). However, a gap in this measure of utility is that it underrepresents the information gained from MAVEs. The aim of this study was to develop an improved quantification metric for MAVE utility. We propose adopting an information content approach that includes data that does not reclassify variants will better reflect true information gain. We adopted an information content approach to evaluate the information gain, in bits, for MAVEs of *BRCA1*,* PTEN*, and *TP53.* Here, one bit represents the amount of information required to completely classify a single variant starting from no information.

**Results:**

*BRCA1* MAVEs produced a total of 831.2 bits of information, 6.58% of the total missense information in *BRCA1* and a 22-fold increase over the information that only contributed to VUS reclassification. *PTEN* MAVEs produced 2059.6 bits of information which represents 32.8% of the total missense information in *PTEN* and an 85-fold increase over the information that contributed to VUS reclassification. *TP53* MAVEs produced 277.8 bits of information which represents 6.22% of the total missense information in *TP53* and a 3.5-fold increase over the information that contributed to VUS reclassification.

**Conclusions:**

An information content approach will more accurately portray information gained through MAVE mapping efforts than by counting the number of variants reclassified. This information content approach may also help define the impact of guideline changes that modify the information definitions used to classify groups of variants.

**Supplementary Information:**

The online version contains supplementary material available at 10.1186/s12859-024-05920-5.

Lea Starita – lstarita@uw.edu.

Brian Shirts shirtsb@uw.edu (Corresponding Author).

## Background

Variant classification guidelines for human disease typically lead to a binary outcome. A variant can either be pathogenic or benign for a human disease. While there are cases where this pathogenic/benign dichotomy may not be suitable, it is appropriate for many genes with clear disease or risk phenotypes. The familiar 5 categories of pathogenic, likely pathogenic, uncertain/VUS, likely benign, and benign add some information about the measure of certainty within the pathogenic/benign dichotomy [1]. Rather than putting all the variants that are thought to be pathogenic in a single category, they are divided into those with higher and lower probabilities of being pathogenic, called pathogenic and likely pathogenic, respectively. The same applies for benign and likely benign variants.

This type of categorization underrepresents the information gained from sources of variant information that come in continuous scales. Similarly, measures of variant-related information utility that look only at transitions of variants into different classification bins also ignore some information that contributes to understanding variant effect. The motivation for this study was to develop and test a measure of variant classification information that overcomes some of the drawbacks of categorical classification. Metrics of certainty about the categorization of binary variables bear a striking resemblance to information theory, where a key measure is the quantification of uncertainty, which is called entropy. By borrowing ideas from information theory, these 5 categories can be replaced by a continuous value, information content, which can precisely convey the certainty of the classification of variants. Specifically, we can convert the probability of pathogenicity to a measure of information content [2, 3]. The use of information theory approaches has similarly been used to plot diagnostic uncertainty in pretest probability and quantify overall diagnostic uncertainty [4–6]. The problem of uncertainty in variant classification is closely adjacent to diagnostic uncertainty and may benefit from the use of similar methods.

In a binary system, information content ranges between 0 and 1 and is often measured in bits. In this context, a bit with a value of 0 means that there is no certainty in the classification of the variant (i.e. it has a 50–50 chance of being pathogenic or benign; alternatively, it has a probability of pathogenicity of 0.5). On the other hand, a bit with a value of 1 means that we are completely certain of the classification of the variant (i.e. the variant is 100% pathogenic or 100% benign; alternatively, it has a probability of pathogenicity of 0 or 1). This would mean that there is complete evidence for classification toward either pathogenicity or benignity.

**Fig. 1:**
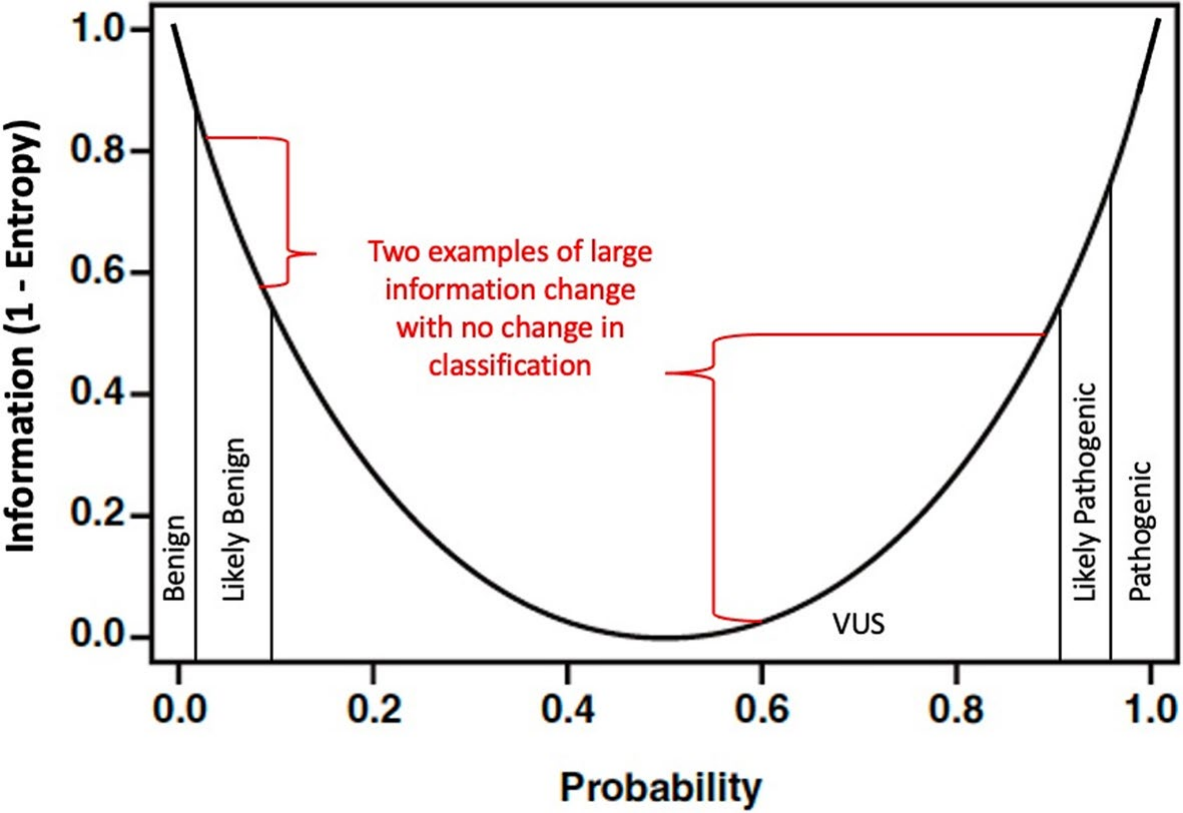
Conversion of probability of pathogenicity to information content, see Vollmer [2]

This shift from discrete categories to a continuous value adds both mathematical and practical benefits. For example, the information content yield of multiplexed assays of variant effect (MAVE) can be more precisely quantified. In a MAVE, a great number of variants are measured for functional effects; however, the clinical value of these studies is usually not reported as the number of variant effects measured, but rather as the number of variants reclassified from VUS to a more certain category. In these studies, many variants do not shift classification categories; thus, a large amount of the information gained is not counted [7, 8] (Fig. 1 modified from Vollmer [2]). An information content framework allows one to quantify information for both variants that are reclassified and variants that are not. In this way, the full information content of the study can be assessed. The full information content of a MAVE, or MAVE information score, can be determined by summing up the changes in certainty for all variants in the study. Changes in information content depend on what information is already present and what is added. If a variant is already clearly classified, adding more evidence does not meaningfully increase the information about that variant. Similarly, the information content framework incorporates evidence that contradicts prior evidence. New evidence supporting pathogenicity for a likely benign variant may decrease the classification certainty and count as negative information. Incorporating all evidence is important for ultimate accuracy, even if it may temporarily decrease the certainty of classification until more evidence arises. These situations may not be reported in the current literature, particularly when there is no shift from one classification to another; using an information framework encourages full reporting of the effects of all evidence on the probability of pathogenicity.

One feature of this method is that the resulting value is easily interpretable and comparable across studies since a value of 1 bit represents 1 variant moved from complete uncertainty (50% chance of either being benign or pathogenic) to complete certainty. This means that if the calculated information content of a study is 30 bits, then it is equivalent to completely classifying 30 variants from being completely uncertain. Note that depending on the study, the same number of bits of information content could be the result of gaining a small amount of information from many variants (as is common for MAVEs, ) or a large amount of information from a small number of variants.

The MAVE information score can be further incorporated into a gene-wide view. A gene can be thought of as a string of information where we know all the information for the reference strand since all positions in the reference should be benign. We may also know that all frameshift and nonsense variants that result in missing functional domains are pathogenic. We do not know all of the information for all possible variant strings. If each possible variant represents a single bit of information (pathogenic or benign), then the number of possible missense variants in a gene is the number of bits a gene needs to be completely classified. This number can be defined as the finish line for completely classifying all possible single nucleotide variants in a gene. If a MAVE’s total information content is divided by this amount, the percentage of all possible information gained is calculated for that study. Alternatively, one can sum the bits for every variant in the gene from a MAVE and all other evidence sources to show overall progress for variant interpretation within that gene.

The major contribution of our work is to show how existing measures underestimate MAVE information content and propose a new, more accurate measure. In this study, we explain the theoretical background for applying information content to MAVEs. We then illustrate these principles with three applications: First, we calculate the proportion of total missense variant information in a gene for several functional studies that have investigated different genes. We then demonstrate that information content can capture much more information than that seen only in changes in classification. We also illustrate how information content calculations can quantify the effect of changing prior probability or altering variant classification guidelines on apparent gene-wide information. The same information framework can also be used to prioritize genes for MAVEs.

## Methods

### Theoretical background

Each specific variant in a gene is either pathogenic or benign for the disease associated with the gene. Each missense variant is assumed to be either pathogenic or benign and thus contain one bit of pathogenicity information. Other variants, such as synonymous variants, may also alter gene function, and truncating variants may be assumed to cause loss of function. While the information content method could be used for non-missense variants, we chose to focus on missense variants, as these are the most commonly assessed variants in MAVEs. For simplicity we will consider that each gene of interest has a single defined gene-disease relationship. Variant classification can be described as gathering information about binary choice. For simplicity, we also ignore issues related to reduced penetrance, as current classification systems have also done. Probability of pathogenicity can be converted to information entropy and then to a measure of information content [2].

Entropy, S, is defined in the equation below where p is the probability of pathogenicity.

S = –p * log2(p) – (1 – p) * log2 (1 – p) * Formula 1*.

Information content is defined as 1-S or equivalently the difference between the maximum value of S, which is 1, and S.

I = 1-S * Formula 2*.

Information content provides a measure of the certainty of an outcome rather than the outcome itself. If there is uncertainty about any bit of information the amount of uncertainty can be expressed as a fraction of a bit of information being available. In this case, a 50% probability of pathogenicity amounts to 0 bits of information. On the other hand, either 0% or 100% probability of pathogenicity amounts to 1 bit of information since no other information could increase the classification (see Fig. 1). For example, if we start with pathogenic moderate evidence, then we can convert that to an odds of pathogenicity of 4.3 [9]. From there, if we assume a prior probability of 0.1, which is commonly done, then we can convert it to a posterior probability of pathogenicity of 0.3246 (using the equation OddsPathogenicity*Prior/((OddsPathogenicity-1)*Prior + 1) also shown in Tavtigian et al. [9]). This posterior probability is equivalent to an entropy of 0.9093 using Formula 1, and an information content of 0.0907 after applying Formula 2. In another example, we could start with benign strong evidence, which can be converted to an odds of pathogenicity of 0.0535. This yields a posterior probability of 0.0059, assuming a 0.1 prior per Tavtigian et al. [9], which is equivalent to an entropy of 0.0522, and finally an information content of 0.9478. Note that the large difference in the information content of the evidence is due in part to the prior of 0.1, which is substantially lower than the zero-evidence prior of 0.5.

Conceptually, thinking of variant classification within the entropy framework as bits of information allows new mathematical transformations. For example, bits of information can be summed and averaged across a larger message or gene. We describe several examples of these applications below. For each of these applications calculations were done with simple formulas in Microsoft Excel. Figures were generated using R statistical software.

### *Application 1: information content of MAVEs*

The total information content generated by several MAVEs was calculated. These MAVEs assessed variants in the genes *BRCA1*, *PTEN*, and *TP53*. Information content gain depends on the information already available from current information, including the prior probability of pathogenicity. For the VUS in Fayer et al. [10], the information content of the MAVEs was calculated as the change in information content for the specific variant with and without the evidence that the MAVE provided. We used data from prior analyses of MAVEs and prior proposed translations of MAVE data to ACMG rules. Data for *BRCA1* were acquired from Supplementary file 2 in Findlay et al. [11]. Data for *PTEN* were acquired from Matreyek et al. [12] (online: http://abundance.gs.washington.edu) and Mighell et al. [13] Table S6. Data for *TP53* were acquired from Table S4 from Fayer et al. [10].

Briefly, MAVE evidence was assigned to ACMG classification criteria as described previously and then converted to odds of pathogenicity as described in Tavtigian et al. [9] and implemented in Fayer et al. [10] This allowed the conversion of categorical evidence, e.g., pathogenic supporting, to a numerical value. The odds of pathogenicity were combined with the prior probability to yield a posterior probability of pathogenicity. This posterior probability of pathogenicity for each variant (see Supplemental Tables S1, S2, and S3 in Fayer et al.) [10] was converted to entropy and information content using formulas 1 and 2. For the *PTEN* and *TP53* MAVEs, information content was calculated using a prior probability of 0.1, as suggested in Tavtigian et al. [9]. Protein variants reported by other MAVE assays were divided into those that can be achieved through a single substitution and those that require more than one DNA change.

### *Application 2: total missense variant information content in a gene*

To calculate the total information content of all missense variants in a gene, we took amino acid sequences for BRCA1, PTEN, and TP53 from the first entry for each respective gene in UniProt [14]. We then examined each amino acid, identified the RNA codons that could code for it, and counted the average number of possible missense, nonsense, and synonymous changes that could be achieved through a single nucleotide substitution. We then used amino acid sequence data for each gene from UniProt [14] and counted the number of possible missense variants for the gene which is equivalent to the total information content of a gene. Some *PTEN* and *TP53* MAVEs include assessments of amino acid changes that require more than one missense variant. Although this method could be used to calculate the information content of those changes, for the sake of simplicity, we restricted our analysis to only single missense variants.

### *Application 3: quantifying the apparent information effect of a classification guideline rule change*

To evaluate how classification guideline rule changes might alter the apparent information gained from applying those rules, we applied each rule across the range of possible prior probabilities and plotted the information content from applying the single rule strength to the prior.

## Results

### Application 1: information content of MAVEs

We used data from prior analyses of MAVEs and prior proposed translations of MAVE data to ACMG rules (see methods) to calculate information content. Evidence criteria were converted to odds pathogenic per Tavtigian et. al. [9]. Then, the posterior probability was determined using standard Bayesian calculations. Posterior probability was then converted to information content (see Methods).

We present two examples variant in *BRCA1* to illustrate this process. These examples only use population frequency and functional data in classification to simplify the examples and to focus on the information content conversion.

*BRCA1* c.5120T > C was classified as a variant of uncertain significance that is absent in population databases (PM2_supporting). Combining the prior probability of pathogenicity of 0.1 with PM2 evidence ((odds*prior)/((odds-1)*prior + 1) derived from Tavtigian et al. [9] would give a posterior probability of pathogenicity of 0.188. This can be converted to an information content of 0.303 by application of Formula 1 and Formula 2 which gives (1-(– 0.188* log2(0.188) – (1–0.188) * log2 (1–0.188))). *BRCA1* c5120T > C has a functional score of -0.143 [10], which is functionally normal and can be used as BS3 evidence, which gives and likelihood of pathogenicity 0.053. Combining this with the population evidence and prior gives a posterior probability of pathogenicity of 0.012. This can be converted to an information content of 0.905 by application of Formula 1 and Formula 2 which gives (1-(– 0.012* log2(0.012) – (1–0.012) * log2 (1–0.012))). The difference in information content that results from incorporating functional data for *BRCA1* c.5120T > C is 0.905 − 0.303, or 0.602 bits of information. The functional data substantially increases the probability that the variant is benign, decreasing uncertainty and increasing information.

*BRCA1* c.5288G > T was classified as a variant of uncertain significance that is absent in population databases (PM2). Before incorporating functional data it has the same probability of pathogenicity (0.188) and information content (0.303) as the prior example. However, *BRCA1* c.5288G > T has a functional score of -1.83 [10], which is functionally abnormal and leads to PS3 evidence associated with a 18.8 likelihood ratio supporting pathogenicity. Incorporating this evidence with prior probability and PM2 evidence, gives posterior probability of pathogenicity of 0.812. This can be converted to an information content of 0.303 from application of Formula 1 and Formula 2 which gives (1-(– 0.812* log2(0.812) – (1–0.812) * log2 (1–0.812))). The difference in information content that results from incorporating functional data for *BRCA1* c.5288G > T is 0.303–0.303, or 0.000 bits of information. Incorporating functional data substantially changes the probability of pathogenicity, swinging the probability of pathogenicity from 0.188 to 0.812, VUS leaning benign or VUS leaning pathogenic, but we are not any closer to certainty about the variant, so the information content does not change.

We summed bits of information across all variants for which MAVE data was available to calculate total information content generated for several MAVEs that assessed variants in *BRCA1*, *PTEN*, and *TP53* [10]. We compared information gain while only considering changes that resulted in VUS reclassification to the information gain from all single nucleotide substitutions reported in MAVE data. Data from MAVEs on amino acid changes that require more than one DNA substitution were excluded from this analysis.

**Table 1:** Information content in bits for reclassifying VUS as presented in Fayer et al. [10], total information content from all single substitutions reported with functional data presented in papers originally listing data, and total possible missense information

	VUS only (Fayer et al.)	Single substitution	Total bits of missense information in gene
*BRCA1*	36.9	813.2	12,351
*PTEN*	10.5	893.6	2721
*TP53*	46.3	160	2571

The *BRCA1* MAVE [11] examined 3893 variants with 2821 functional, 249 intermediate, and 823 showing loss of function. A functionally normal classification was considered strong benign evidence and loss of function was considered strong pathogenic evidence. Conversion of this evidence to posterior probability using prior probability of 0.1 and then to information content resulted in 813.2 bits of information gained by the *BRCA1* MAVE.

The *PTEN* MAVEs [12, 13] examined 8198 variants for effects on protein abundance and examined 7657 variants for activity. The number of overlapping variants was 7639, of which 4811 had combined scores that were considered strong pathogenic evidence and 303 which were considered benign supporting evidence. Conversion of this evidence to posterior probability using prior odds of 0.1 and then to information content yielded a total information content of 893.6 bits of variant classification information added by the study for changes possible through single missense substitutions.

Four *TP53* MAVEs were combined and used to train a naïve Bayes classifier and make predictions on 7893 variants with scores in each of the four assays [10, 15, 16]. The Bayes classifier predicted 5070 as normal and 2823 as abnormal. These were assigned weights of benign moderate and pathogenic strong evidence, respectively. Conversion of this evidence to posterior probability using prior odds of 0.1 and then to information content yielded 160.0 bits of variant classification information for changes possible through single missense substitutions.

Comparing reports that included only VUS reclassified to our method, which included all the classification information generated by several MAVEs, the reported information content increased 22-fold for the *BRCA1* assay, 85-fold for the *PTEN* assays, and 3.5-fold for the *TP53* assays. (See Table 1)

### Application 2: total missense variant information content in a gene

**Fig. 2:**
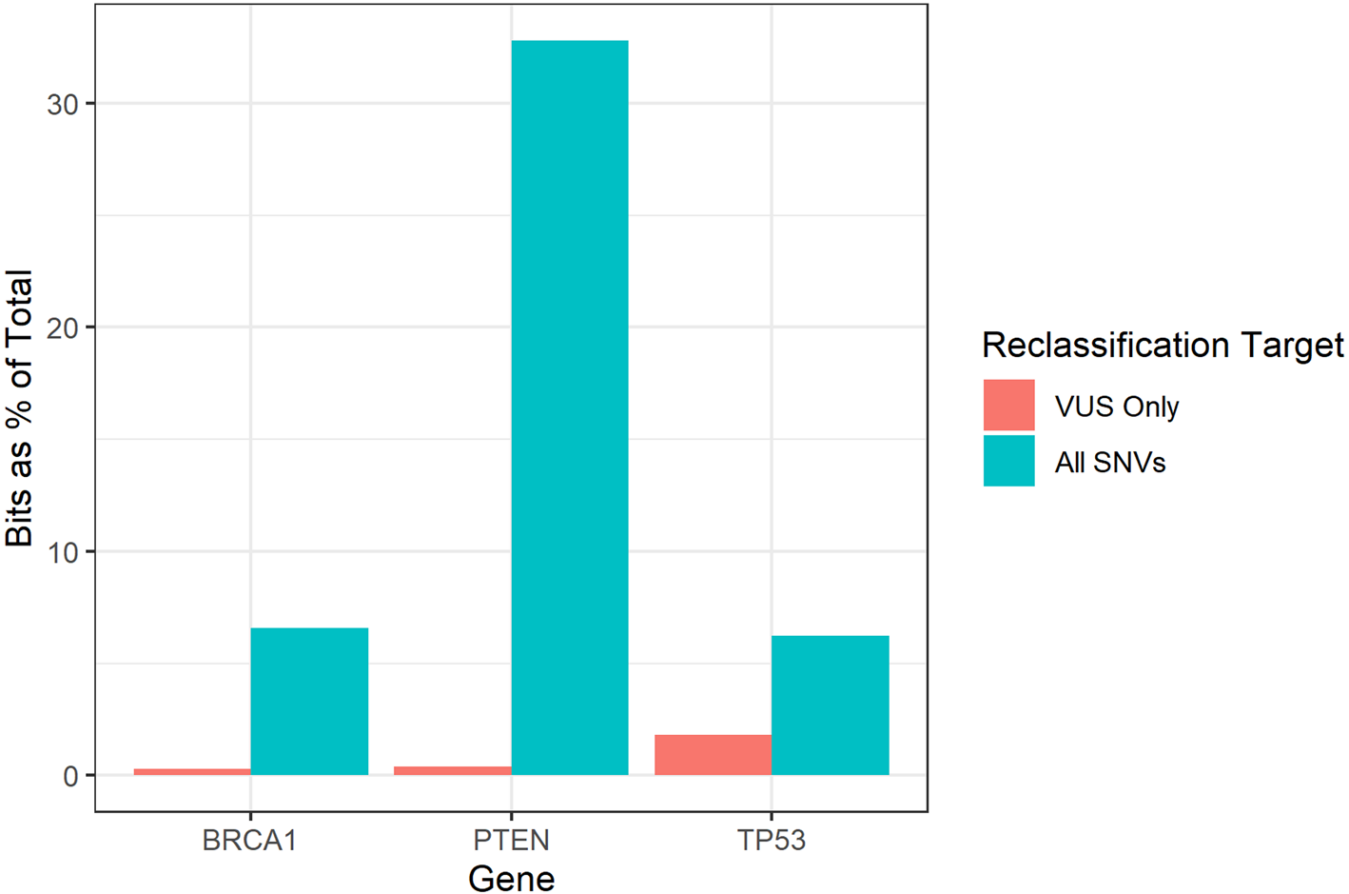
Percent of total information found by each study. A plot of the percentage of total information found by each of the studies

The total missense information content of a gene can be calculated by counting the number of possible missense variants in a gene. Since each variant represents one bit of information, the total missense information content of a gene, in bits, is the number of possible missense variants. These values were 12,351; 2721; and 2571 for *BRCA1*, *PTEN*, and *TP53* respectively. The MAVEs generated 6.7%, 32.8%, and 6.2% of the total possible single-substitution variant classification information for *BRCA1*, *PTEN*, and *TP53* respectively. (Fig. 2)

### Application 3: quantifying the apparent information effect of a classification guideline rule change

**Fig. 3:**
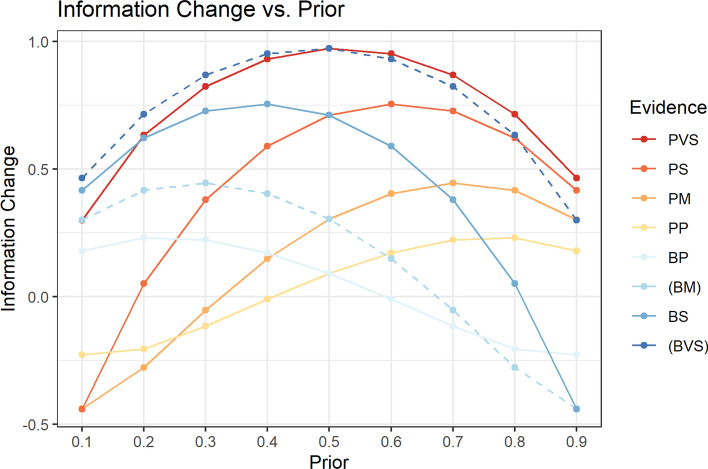
Plot of the information change for the different types of evidence across different prior probabilities. Information content loss for pathogenic evidence occurs at lower prior probabilities since these priors contain high information content for benign interpretation. The incorporation of pathogenic evidence for a variant with a low prior moves the probability in the pathogenic direction and toward greater uncertainty. The same effect occurs for benign evidence with a high prior probability of pathogenicity since incorporation of benign evidence will reduce the probability moving classification toward a more uncertain class, thus reducing information content. The listed evidence categories are pathogenic very strong(PVS), pathogenic strong(PS), pathogenic moderate(PM), pathogenic supporting(PP), benign supporting(BP), benign moderate(BM), benign strong(BS), and benign very strong(BVS). Note that benign moderate and benign strong are not currently approved categories but are listed in parentheses for completion.

For well-established functional studies the 2015 ACMG-AMP guidelines recommend using the evidence codes PS3 and BS3 indicating strong evidence for or against pathogenicity, respectively [17]. However, different levels of evidence have been proposed for MAVEs that meet stronger or weaker validation criteria [7, 18–21]. If the variant classification guidelines or strength of evidence criteria change, the changes have an effect on the apparent information for any variant that is impacted by the guideline change. For example, evidence against pathogenicity for *PTEN* MAVEs is considered supporting evidence primarily because there are very few established benign *PTEN* variants to use in validation. If the amount of validation data increases the evidence generated by the MAVE may change. This would result in an apparent change in information content for many variants. Similarly, if ACMG-AMP committees or ClinGen VCEPs decide to refine the level of evidence assigned to a specific rule, there are many variants for which the apparent information content would change. We evaluated how applying single ACMG-AMP evidence levels with different priors using the Tavtigian et al. [9] Bayesian framework would result in different levels of evidence (Fig. 3). This analysis illustrates how the greatest information gains always occur when the prior probability is 0.5. Differences between points on the same vertical prior line show how shifting evidence assignment will change apparent information. This also illustrates that applying benign evidence to a variant with a high prior probability results in an apparent information loss from the increase in uncertainty or increasing entropy (all points with information change below 0 as plotted on the y-axis). There is a similar result when pathogenic evidence is applied to a variant with a low prior probability.

Figure 3 plots how different evidence levels combined with prior probabilities result in different amounts of apparent informatio

## Discussion

Currently, the clinical utility of MAVEs is measured as the number of individual variants classified or reclassified from variants of uncertain significance (VUS) to other classifications. Under current practice, a study that reclassifies a small number of variants may be perceived as more useful than a study that provides evidence for many variants but not enough for any one variant to be reclassified. An information content framework for evaluating MAVEs will provide a more accurate measurement of the full contribution of functional data evidence. There are many smaller functional studies that do not use high throughput MAVE methods, the information content framework is predicted to provide a more accurate measure of evidence for such smaller functional studies as well. We have shown that reporting only changes in classification undervalues the information yield of a MAVE, often by more than an order of magnitude.

In this study we focused on the change in relative information content, but the absolute information content of a study can be quantified by calculating information content using a prior of 0.5 which assumes no prior information. This effectively eliminates any conflicting information and thus only yields positive information. With relative information content a study can seemingly provide negative information for individual variants due to new evidence conflicting with the prior evidence (see Fig. 3). While the idea of negative information content may seem odd at first glance, intuitively it makes sense that new conflicting information leads to a loss of certainty. It can also be thought of as the new information cancelling out previous information. This is evident in the *TP53* analysis where variants yielded 0.3 bits if they were functionally normal and − 0.44 bits if they were functionally abnormal assuming a 0.1 prior. Since there were 1181 functionally abnormal variants, this led to a low total information content for the MAVE. If we instead assumed a prior of 0.5, the variants would yield 0.3 bits if they were functionally normal and 0.71 bits if they were functionally abnormal. This would result in a total of 1518.61 bits of information instead of the 160 bits listed in Table 1.

In addition, an information content framework allows gene-level reporting of information in that it enables quantification of the total percentage of information provided by a MAVE for a given gene. The ability to quantify information as a percentage of a whole at the gene level has several benefits: it defines the variant information that exists, it can effectively illustrate the proportion of total information a specific MAVE has produced, and it can accurately illustrate what proportion of information remains missing for a gene or a variant. In the context of relative information, it can help prioritize future MAVEs by showing genes where a small proportion of total information exists and where efforts are likely to lead to a large increase in relative information.

Finally, understanding how classification rules influence apparent information will help committees that arbitrate guidelines for variant classification to quantify the effect of changing any specific rule more accurately across many variants in a gene. In addition to scrutinizing the effect on a few variants, summing or averaging the change in information over all variants can provide a global assessment of the information change implied by the change in the strength assigned to evidence rules. The goal of classification guidelines should be to accurately quantify the information from different sources. Knowing how specific individual variants are classified is a clinical imperative; however, using a variant information framework shows how high-throughput methods and rule modifications can change the entire information landscape of the gene.

The information content approach has limitations similar to those any measure of information content when there is uncertainty about the real message; when we do not know the final outcome, we can only estimate the true amount of information about variant classification that is delivered by any MAVE. Another limitation is that this method relies on a Bayesian framework for variant classification that has not been universally implemented. To most accurately understand the added information coming from a new source, it is important to be able to quantify the existing information. That information is currently expressed as a broad categorical classification. At present broad variant classifications are converted to odds ratios and then to probabilities then to bits of information. The forthcoming harmonized points system that is correlated with Bayes values will provide more accuracy about prior information and simplify Bayesian conversions. A strength of the information content method proposed in this paper is that it has minimal complexity, calculations can be done at scale quickly with any statistical software.

In future work, we will apply our information content framework to additional MAVEs, and we encourage others to do so as well. A similar method could also be used to apply information content strategies to in silico variant data or to both MAVE and in silico data when used together. Variant classification committee should evaluate changes in information content with a classification rule change across many variants to determine whether such changes appropriately reflect expected information content.

## Conclusions

Counting the number of VUS reclassified is a dramatic underestimate of the information content of MAVEs. Variant reclassification information reported as a change in information content provides a more accurate representation of the true information gained. We believe that incorporating an information content framework when presenting reclassification evidence will lead to a better understanding of the value of different classification projects and, in the end, better classification of variants. It will also allow quantification of how much information is missing for different genes allowing prioritization of genes with less relative knowledge. Reporting information content will better allow geneticists to understand the contributions of high-throughput information sources and guideline rule changes more wholistically. In turn, better classification leads to better clinical validity and utility of genetic testing and genetic risk assessment.

## Supplementary Information

Supplementary Material 1,

## Data Availability

No datasets were generated or analysed during the current study.
