# Supplementary material for: Assigning credit where it is due: an information content score to capture the clinical value of multiplexed assays of variant effect
Source: BMC Bioinformatics. 2024 Sep 6;25:295. doi: 10.1186/s12859-024-05920-5 (PMC11380199; doi:10.1186/s12859-024-05920-5)
Supplement: Supplementary file 1 — Supplementary Material 1, [file 12859_2024_5920_MOESM1_ESM.docx]

**Supplementary information for Ranola et al. “Assigning credit where it's due: An information content score to capture the clinical value of Multiplexed Assays of Variant Effect”**

Several supplementary files are referenced in the manuscript.

Supplementary file 2 in Findlay et al. 2018 can be found through the following reference: Findlay GM, Daza RM, Martin B, Zhang MD, Leith AP, Gasperini M, et al. Accurate classification of BRCA1 variants with saturation genome editing. Nature. 2018;562:217–22.

Supplemental Tables S1, S2, S3 in Fayer et al. can be found through the following reference: Fayer S, Horton C, Dines JN, Rubin AF, Richardson ME, McGoldrick K, et al. Closing the gap: Systematic integration of multiplexed functional data resolves variants of uncertain significance in BRCA1, TP53, and PTEN. Am J Hum Genet. 2021;108:2248–58
